# Anatomy for Cardiac Electrophysiologists - A Practical Handbook. Authors: S. Yen Ho, Sabine Ernst. Cardiotext Publishing, LLC

**Published:** 2013-11-15

**Authors:** Johnson Francis

**Affiliations:** Senior Consultant Interventional Cardiologist, Baby Memorial Hospital, Calicut, Kerala, India

**Keywords:** Cardiac Anatomy, Electrophysiology

Thorough knowledge of the heart and its conduction system is quint essential for the cardiac electrophysiologist. *Anatomy for Cardiac Electrophysiologists - A Practical Handbook* is a well illustrated book in this context. It has packed a whole lot of useful information into just 250 pages. Anatomical photographs are supplemented by histological sections when needed. All imaging modalities right from simple fluoroscopy for catheter positions to echocardiography and three dimensional computed tomographic images are available. The book comes with a foreword from none other than Melvin M Scheinman, one of the pioneers of cardiac electrophysiology.

The book has twelve chapters, divided into four sections. The initial four chapters constitute an overview of anatomy and imaging in 64 pages. The section includes chapters on general cardiac anatomy, the neighboring structures indicating the chance of collateral damage, an overview of various imaging modalities and the anatomy relevant to standard electrophysiology catheter positions.

The second section on the atria has another four chapters running into 114 pages. The chapters describe the electrical anatomy and accessory pathways, aspects of right atrium relevant to supraventricular tachycardias and notes on atrial septum and trans septal access. Left atrial anatomy with special reference to pulmonary vein related procedures for atrial fibrillation are also detailed.

Third section is on the ventricles and their malformations, divided into three chapters of 52 pages in total. One chapter each is devoted to the right ventricle, left ventricle and congenital malformations. The final section with one chapter is on pitfalls and troubleshooting.

This wonderful book integrates anatomy with all modern imaging modalities being used in the field of cardiac electrophysiology ranging from 3D-maping to 3D-echocardiography and 3D-CT reconstruction. This book will be of great help to the novice in electrophysiology in learning the procedures as well as to the practicing electrophysiologist to brush up.

